# Positive Effect of Visual Cuing in Episodic Memory and Episodic Future Thinking in Adolescents With Autism Spectrum Disorder

**DOI:** 10.3389/fpsyg.2019.01513

**Published:** 2019-07-09

**Authors:** Marine Anger, Prany Wantzen, Justine Le Vaillant, Joëlle Malvy, Laetitia Bon, Fabian Guénolé, Edgar Moussaoui, Catherine Barthelemy, Frédérique Bonnet-Brilhault, Francis Eustache, Jean-Marc Baleyte, Bérengère Guillery-Girard

**Affiliations:** ^1^Normandie Université, UNICAEN, PSL Universités Paris, EPHE, INSERM, U1077, CHU de Caen, Neuropsychologie et Imagerie de la Mémoire Humaine, Caen, France; ^2^Service de Psychiatrie de l’Enfant et de l’Adolescent, CHU de Caen, Caen, France; ^3^UMR 1253, iBrain, Université de Tours, INSERM, Centre Universitaire de Pédopsychiatrie, CHRU de Tours, Tours, France; ^4^Service de Psychiatrie de l’Enfant et de l’Adolescent, CHI de Créteil, Créteil, France

**Keywords:** autobiographical memory, episodic memory, visual cues, sensory details, autism

## Abstract

Cognitive studies generally report impaired autobiographical memory in individuals with autism spectrum disorder (ASD), but mostly using verbal paradigms. In the present study, we therefore investigated the properties of both past and future autobiographical productions using visual cues in 16 boys with ASD and 16 typically developing (TD) participants aged between 10 and 18 years. We focused on sensory properties, emotional properties, and recollection, probing past and future productions for both near and distant time periods. Results showed that the ASD group performed more poorly than controls on free recall for recent periods, but performed like them when provided with visual cues. In addition, the ASD group reported fewer sensory details than controls and exhibited difficulties in the experience of recollection for the most remote events. These data suggest a combination of consolidation and binding deficits. Finally, our findings reveal the relevance of using visual cues to probe autobiographical memory, with possible perspectives for memory rehabilitation.

## Introduction

Autism spectrum disorder (ASD) is a neurodevelopmental disorder, characterized by deficits in social communication, with restricted and repetitive behaviors. There is growing evidence that people with ASD have atypical memory functioning (Bowler et al., 1997), even if their language skills are intact. Difficulties include, among others, impairment of autobiographical memory (AM). AM is a very long-term memory of personal knowledge and events related to individuals’ own lives that are accumulated from a very early age. AM allows individuals to build an identity based on a feeling of continuity (Conway, 2005; Bon et al., 2012).

Current cognitive models of AM distinguish between a semantic component pertaining to general personal knowledge or facts, and an episodic component relating to personal events. This episodic component relies on the ability to remember past experiences (i.e., episodic autobiographical memories) and to imagine possible future experiences (episodic future thinking) (Tulving, 1985). Both episodic memories and projections involve autonoetic consciousness, namely the ability to project our states of self into the past, present or future to maintain self-continuity. This mental time travel allows individuals to re- or pre-experience personal events associated with their original context, giving individuals a feeling of (re)living these events. To evoke episodic events, sufficient phenomenological details (i.e., feelings, emotions, sensory details such as colors, sounds, smells, tactile feelings) must be stored in memory, as they serve as retrieval cues. More specifically, episodic future thinking or projection involves imagining oneself in the future to *pre-experience* a possible scenario (Atance and O’Neill, 2005). This projection is supported in part by episodic memory oriented toward the past (Suddendorf and Corballis, 1997; Wheeler et al., 1997). Moreover, remembered personal events and envisioned future plans have been found to share a common brain network (Viard et al., 2011; D’Argembeau, 2015). This network is thought to support common constructive thought processes that allow for the retrieval and flexible combination of stored information to reconstruct past experiences and construct novel future ones. Besides constructive and executive processes, AM involves a broad range of cognitive processes, ranging from perception (Gottfried et al., 2003) to more integrative processes. Some of these are preferentially related to the self (self-concept: Howe and Courage, 1997; theory of mind: Perner and Ruffman, 1995; Welch-Ross, 1997) and social events (Nelson, 1993), while others refer to narrative abilities (Kleinknecht and Beike, 2004). Hence, the maturation of these cognitive processes during childhood and adolescence supports AM development (Nelson and Fivush, 2004; Bauer et al., 2007; Piolino et al., 2007; Picard et al., 2009).

In ASD, both children and adults produce fewer specific memories and projections, characterized by reduced specificity, elaboration and episodic coherence. The content of these memories is also more semantic (e.g., general or repeated event) than episodic (Bon et al., 2012; Crane et al., 2012, 2013; Terrett et al., 2013; Goddard et al., 2014; McDonnell et al., 2017). Ciaramelli et al. (2018) recently reported that providing a series of standardized questions (e.g., “Where did this event take place”) does not seem to increase performance, either for past recollection or for future thinking. Similarly, difficulty retrieving specific memories is observed in children and adolescents with ASD, with poorer access to the remote past (8- to 17-year-olds; Goddard et al., 2014), and impaired episodic future thinking (8- to 12-year-olds; Terrett et al., 2013). Children with ASD also have greater difficulty recalling their own activities than typically developing (TD) children (Millward et al., 2000). However, differences may be observed between children and adults with ASD. For example, discourse analysis has shown that children with ASD aged 6–14 years produce fewer past narrative details, as well as fewer emotional (e.g., *happy*, *scared*), cognitive (e.g., *thought*, *believed*), and sensory (e.g., *seen*, *heard*) terms than TD children (Brown et al., 2012). This difference is more pronounced for remote life events than for recent ones for children aged 5–17 years (Bruck et al., 2007; Brown et al., 2012; Goddard et al., 2014) or future thinking (Terrett et al., 2013). On the contrary, results obtained in adults show that sensory references are more frequent in ASD than in TD for self-defining memories (Crane et al., 2010) and early childhood events (Zamoscik et al., 2016). Hence, some sensory details may be more salient than other features and contribute to the structure of AM in adulthood. This heterogeneity highlights the importance of exploring changes between childhood and adulthood, by focusing on the adolescence period.

The impairment of AM in ASD can be interpreted according to different cognitive theories. First, the theory of mind deficit resulting in difficulty recognizing one’s own psychological states and understanding of the self (Williams, 2010) may impact the narration of episodic events (Losh and Capps, 2003; Goldman, 2008; McCabe et al., 2013; Kristen et al., 2014). Second, a detail-focused perceptual style, which refers to perception theory, or the *weak central coherence* evoked by Happé and Frith (2006), may also have a significant impact on the properties of autobiographical memories. Temple Grandin, a woman with high functioning ASD, reported in her 2006 book *Thinking in Pictures* (Grandin, 2006) that the visual modality is ubiquitous in her daily life:

“I translate both spoken and written word into full-color movies, complete with sound, which run like a VCR tape in my head… [I] see the words in pictures … I have a video library… When I recall something I have learned, I replay the video in my imagination. The videos in my memory are always specific … My imagination works like the computer graphics programs … When I do an equipment simulation in my imagination or work on an engineering problem, it is like seeing it on a videotape in my mind. I can view it from any angle, placing myself above or below the equipment and rotating it at the same time… I create new images all the time by taking many little parts of images I have in the video library in my imagination and piecing them together… Unlike those of most people, my thoughts move from video-like, specific images to generalization and concepts. For example, my concept of dogs is inextricably linked to every dog I’ve ever known. It’s as if I have a card catalog of dogs I have seen, complete with pictures, which continually grows as I add more examples to my video library.”

She describes her visual memory as a collection of personal *photographs* of her own life, which has a direct impact on the formation of visual representations of semantic concepts. Moreover, she is able to take different perspectives but, as suggested by her testimony, these tend to be field perspectives with egocentric navigation. This was experimentally corroborated by Ring et al. (2018). Hence, visual autobiographical memories may be very specific and detailed but more fixed than those of TD people.

Third, the AM deficit in ASD may result from difficulty mentally assembling the details that form the experience (e.g., *episodic simulation*; Schacter et al., 2012) and elaborating the context of this experience (e.g., *scene construction*; Hassabis and Maguire, 2007). Scene construction relies on visual imagery which involves the mental generation and maintenance of a single element and the binding of all the properties of the event (e.g., objective and subjective details). Poorer scene construction is consistent with the impaired binding processes observed in ASD (Bowler et al., 2011; Lind et al., 2014a).

Most studies reporting difficulties with AM were conducted using verbal paradigms that elicit narrative abilities (Goddard et al., 2007; Crane and Goddard, 2008; Crane et al., 2009, 2012). Since these narrative abilities are impaired in ASD, solely using language to investigate AM may bias the assessment of memory properties. Most of the studies that have reported an AM impairment in ASD used questionnaires or a fluency task. However, individuals with ASD performed just as well as controls when other methodologies were used. No differences were observed with the use of a sentence completion test that indexes memory retrieval (Crane et al., 2013), or yes–no questions (Bruck et al., 2007), when the recall test was written rather than oral (Crane et al., 2012) or when the cue words were high in imageability (e.g., *letter* vs. *permission*) (Crane et al., 2012). All these tasks provide cues or support at retrieval. These observations are in line with the *task support hypothesis* that emphasizes the role of retrieval support in improving AM productions (Bowler et al., 2004).

Hence, and as suggested by Temple Grandin’s testimony, pictures could be a valuable tool for studying AM, by providing a visual aid to overcome the language constraints associated with the free recall paradigm. Therefore, pictures would constitute a more appropriate mean of testing the properties of episodic memories in ASD. In addition, these visual supports would provide an opportunity to test different kinds of properties, including sensory details, and investigate the possible impact on AM of the impairments in sensory processing observed in ASD (Stevenson et al., 2014).

The main aim of the present study was to investigate the properties of episodic memories and future thinking in high-functioning adolescents with ASD using visual cues. We focused on the sensory and emotional properties and the quality of the experience of recollection associated with autobiographical productions for four time periods: two in the past (i.e., yesterday and last summer vacation) and two in the future (i.e., tomorrow and next summer vacation). First, given the known retrieval deficit in ASD and possible difficulties in scene construction, we predicted that free recall performance would be impaired, but performance would normalize when visual cues were provided. We added a general neuropsychological assessment focusing on cognitive functions involved in AM retrieval, i.e., executive functions, short-term memory, and verbal episodic memory, to discuss our results. Based on the cognitive profile of ASD, we expected to find baseline differences in verbal episodic memory, planning and short-term memory. Second, given the perceptual bias reported in ASD (Mottron et al., 2003) and the frequent references to sensory details reported by adults with ASD (Crane et al., 2010), we predicted that participants would exhibit an atypical pattern of performance concerning sensory properties, with a probable focus on some perceptual modalities to the detriment of others. Third, given the well-known difficulty with emotion processing and reduced recollection capacity in ASD (Gaigg, 2012), we expected participants to perform poorly on emotion and recollection assessment.

## Materials and Methods

### Participants

Participants were 16 boys aged 10–18 years (mean = 13.4 years, *SD* = 2.4) (Table 1). They were recruited through autism resource centers in Caen and Tours in France. The recruitment started prior to the 2013 publication of DSM5, hence participants had all been diagnosed with verbally and intellectually high-functioning autism or Asperger’s syndrome according to DSM-IV (American Psychiatric Association [APA], 2000) criteria. The diagnosis was established by experienced professionals using the Autism Diagnostic Interview-Revised (ADI-R; Lord et al., 1994) and/or Autism Diagnostic Observation Schedule (ADOS; Lord et al., 1989). The ADI-R is a detailed semi-structured interview of parents about their child’s developmental history and autism symptoms that yields ratings for reciprocal social interaction, language and communication, and restricted repetitive behaviors. The ADOS is also a semi-structured interview and is a standardized assessment of social interaction, communication, play and imaginative use of materials. Participants with ASD were compared with 16 TD controls matched for age, sex, and scores on the Perceptual Reasoning Index (PRI) and Verbal Comprehension Index (VCI) of the fourth version of the Wechsler Intelligence Scale for Children (WISC-IV; Wechsler, 2005). These two indices were calculated according to performances on four WISC-IV subtests: Block Design and Matrices for PRI, and Vocabulary and Similarities for VCI. They allowed us to ensure that participants had no general impairment of language comprehension or perceptual abilities. TD adolescents were recruited from several French schools. Brief interviews ensured that none of the participants met the exclusion criteria: history of previous neurological disorders or psychiatric illness (other than ASD in the ASD group), a first-degree relative with ASD in the TD group, head trauma, current psychoactive medication, intellectual disability, and learning disabilities. Families were given a comprehensive description of the research. The study was approved by the relevant ethic committees, and written consent was obtained from all the participants (and their parents, in the case of minors), in line with committee guidelines.

**Table 1:** Mean ages and cognitive data for the ASD and TD groups.

	ASD (*n* = 16)		TD (*n* = 16)		Group differences *p*-value and effect size
	Mean	*SD*	Mean	*SD*	
Age (in years)	13.4	2.4	13.0	2.0	*p* = 0.54, η^2^ = 0.01
PRI	101.3	17.7	109.4	16.3	*p* = 0.22, η^2^ = 0.05
VCI	108.3	21.4	116.1	14.7	*p* = 0.13, η^2^ = 0.07
Short-term memory and executive functions					
Tower of London					
Success at first attempt	7.1	1.5	7.9	1.5	*p* = 0.03^∗^, η^2^ = 0.14
Total number of trials	19.7	3.7	19.5	4.9	*p* = 0.18, η^2^ = 0.06
Stroop	33.2	10.8	27.1	10.1	*p* = 0.24, η^2^ = 0.08
Visuospatial span	6.1	1.7	5.9	1.2	*p* = 0.87, η^2^ = 0.001
Verbal span	5.9	1.1	6.1	1.3	*p* = 0.61, η^2^ = 0.009
Episodic memory					
Immediate recall	22.4	9.4	28.9	6.3	*p* = 0.03^∗^, η^2^ = 0.14
Delayed recall	21.2	9.3	26.8	6.1	*p* = 0.09, η^2^ = 0.09
Recognition	11.6	2.4	13.4	0.9	*p* = 0.01^∗^, η^2^ = 0.18
Personal semantic knowledge					
Acquaintances	5.8	0.5	5.9	0.5	*nd*
School life	5.9	0.1	5.9	0.1	*nd*
Famous names	5.9	0.3	6.0	0.0	*nd*

*^∗^Significant differences observed between the ASD and TD groups. nd, not done owing to a ceiling effect; PRI, Perceptual Reasoning Index; VCI, Verbal Comprehension Index; ASD, participants with autism spectrum disorder; TD, typically developing participants.*

### General Cognitive Assessment

Each child also underwent a neuropsychological assessment focusing on the cognitive abilities involved in AM production (Picard et al., 2009). This assessment included tests of five executive and memory functions: inhibition (Stroop test; Albaret and Migliore, 1999), planning (Tower of London; Lussier et al., 1998), verbal short-term memory (forward digit span, WISC), visuospatial short-term memory (Forward Corsi blocks; Pagulayan et al., 2006), and verbal episodic memory (story recall from Children’s Memory Scale; Cohen, 2001). Picard et al. (2009) found that these cognitive abilities were involved in the production of autobiographical memories in childhood (6–11 years).

Finally, all participants underwent a brief investigation of personal semantic knowledge, in order to exclude a possible major deficit that might interfere with the AM task. This consisted of a questionnaire coupled with visual cues about general personal information on three different topics, adapted from Piolino et al. (2007)’s methodology. Questions concerned acquaintances, school life, and personally relevant famous names (e.g., heroes, stars, etc.). The maximum score was 6 for each of these categories.

### From Past to Future Task

**FIGURE 1:**
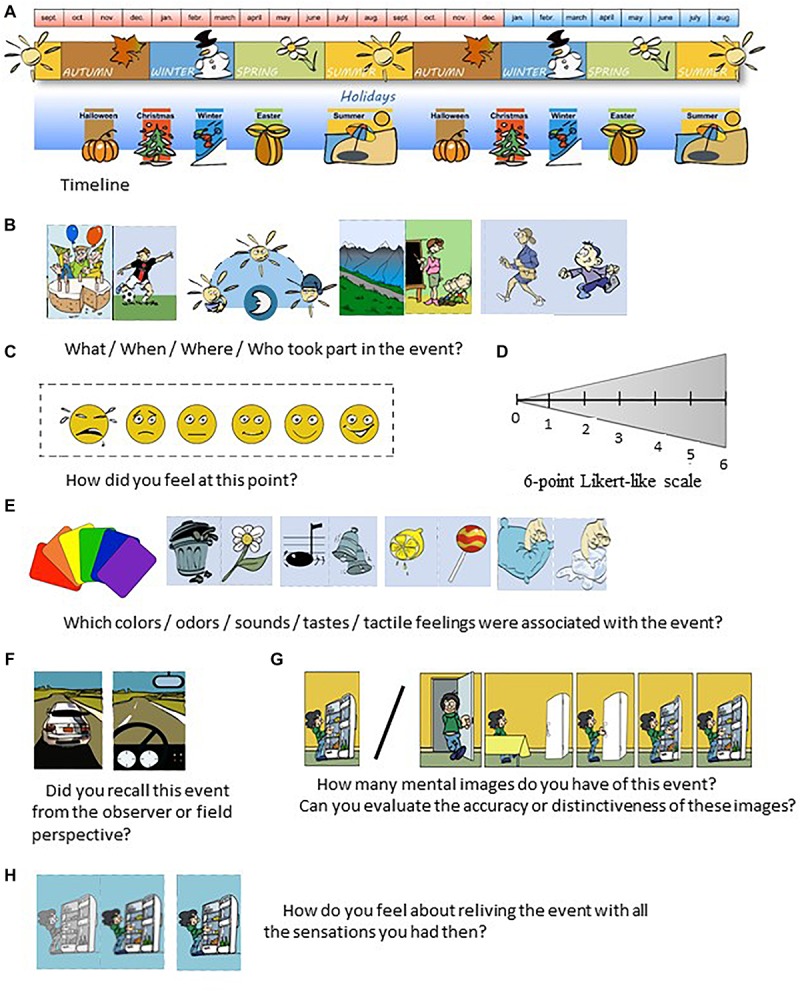
From past to future task. This task explored specific past personal events that had occurred either the day before (recent past) or during the previous summer vacation (remote past), as well as projections to the next day (near future), or forthcoming summer vacation (distant future). First, participants were provided with a visual timeline and asked to point to the current day, to ensure that they were oriented in time **(A)**. Second, participants were asked to describe a memory or future event for each period with as many details as possible. If, after 1 min, any of the participants were not able to provide the different types of contents associate with an episodic event, they were helped with visual cues for each of the following components: what, how, when, where, who **(B)**, emotions **(C)**, 6-point Likert scale **(D)**, perceptions (e.g., color; **E**), perspective (field or observer; **F**), mental imagery **(G)**, and reliving **(H)**.

This task explored specific past personal events and future thinking for the day before (recent past), last summer vacation (remote past), next day (near future), and forthcoming summer vacation (distant future). For each period, visual cues were provided to support production (Figure 1). All responses were directly manually transcribed by the interviewer. The interviewer had a grid for coding each personal event that was reported (free recall and cued recall of personal event). All other responses were directly coded by the participants themselves.

### Visual Cues

Questions were illustrated with drawings that provided a timeline and visual cues for detailing personal events, contents and perceptions (i.e., colors, smells, tactile feelings, sounds, tastes). Contents could refer to temporal situations, spatial locations (e.g., home, school, beach, etc.), modes of transport (e.g., car, plane, train, etc.), activities (e.g., video games, football, musical instrument, etc.) and people present (e.g., parents, children, etc.). All the pictures were drawn by a professional illustrator who ensured that each type of content was included. For example, for the *who* content, there was a person of every age (i.e., children, adults, and older adults) and gender. In addition, five types of perceptions were illustrated with drawings. For example, colors were associated with a color chart, while smells were indicated with a trash can or a flower; sounds with a musical note or bell; tastes with a lemon or a sweet; and tactile feelings with a finger placed on a pillow (mushy) or on ice (cold) (Figure 1E). Each question included explanations of the properties being tested (e.g., “Did you have tactile feelings? Did you touch something soft like cotton wool, cold like ice, mushy like a pillow, hard like wood, wet like water, or painful like a hedgehog?”). Participants repeated the property when they selected the drawing that supported their autobiographical production (e.g., “I touched something soft…”). This procedure was applied to all visual cues.

### Procedure

Each participant was asked to produce descriptions of memories or projections with as many details as possible, focusing on the past (i.e., one event that happened yesterday and one last summer vacation) and the future (i.e., one event that could happen tomorrow and one next summer vacation). These questions allowed us to manipulate orientation (past vs. future) and temporal distance, either close (yesterday or tomorrow) or remote (last or next summer vacation). For past events, participants were instructed to remember real events that had happened to them (e.g., “Can you remember something that happened to you yesterday? I want you to recall it with plenty of details, as if you were reliving this event, and your description has to allow me to imagine this event too”). For future events, participants were instructed to imagine an event that could happen in their lives or else was completely invented (e.g., “Can you imagine what you might do tomorrow, either something planned or completely new, but I want you to imagine what could happen with plenty of details, as if you were living this event, and your description has to allow me to imagine this event”). If 1 min went by without an answer, the interviewer gave the children an open-ended prompt (e.g., “What else can you remember?”). If they were still not able to provide different contents associated with an episodic event, after a further minute, they were helped with visual cues for each of these components. Cues concerned activities (*what*), temporal situation (*when*), spatial location (*where*), course of the event (*how*), and people present (*who*) (Figure 1A,B). Episodic free recall and cued recall (with visual cues) were each scored out of 5, with 1 point per type of content: what (theme), when (e.g., beginning, middle or end of the month; morning, afternoon or evening), where (which city and where in that city; e.g., home, garden, beach), how (three different details; e.g., perception, feeling, activity, script), and who (participants). Scoring was performed separately by the interviewer and a psychologist until a consensus was reached (Table 2).

**Table 2:** Episodic memory paradigm, variables, and scoring.

**Personal event**^∗^		
What, when, where, how, who	Free recall	/5
What, when, where, how, who	Visual cued recall	/5
**Emotional feeling**		
	Valence	/6
	Arousal	/6
**Sensory details**		
	Details	No.
Color	Importance	/6
	Details	No.
Sound	Importance	/6
	Details	No.
Smell	Importance	/6
	Details	No.
Touch	Importance	/6
	Details	No.
Taste	Importance	/6
**Mental imagery**		
	Details	/6
	Accuracy	/6
**Perspective**		
	Field perspective or	3
	Field/observer perspective	2
	or Observer perspective	1
Subjective recollection		/6
Personal relevance		/6
Frequency of evocation		/6
Wish for it to happen^#^		/6
Probability of occurrence^#^		/6

*^∗^Coded by two persons: the interviewer and the psychologist. All other measures were directly coded by participants. ^#^Variables for future periods only. No, number.*

Next, we asked participants about the properties of each event. Participants rated their own productions. First, we asked them to rate the emotional feeling associated with the event on a 6-point Likert-like scale featuring smiley faces ranging from very sad to very happy (e.g., “I was happy to do this, so I choose the fifth smiley”; Figure 1C). They also rated the level of emotional arousal on a triangular ruler, again with a 6-point Likert-like scale along each side (e.g., “I was happy to do this, but not very excited, so I rate it 2 on the scale”; Figure 1D). The Likert-scale was used for all the following questions. Participants were then asked to provide sensory details (i.e., colors, sounds, smells, tactile feelings, tastes; Figure 1E), and indicate the importance of each one in their memories or future thinking, using the same 6-point triangular ruler (e.g., “Which colors do you remember being associated with your memories? What was the intensity of each one?”). In the final part of the questionnaire, we collected other information. One question concerned the perspective from which they had relived the event: either their own (field perspective, scored 3/3), that of an observer (observer perspective, scored 1/3), or alternating between the two (scored 2/3) (Figure 1F). Another question assessed the mental imagery associated with the personal event, asking participants whether they could visualize the personal event in terms of the number of images (e.g., “When you think about this event? How do you see it? Please rate it on a scale from 0 (*No image*) to 6 (*Lot of distinctive images*)”; Figure 1G) and accuracy (e.g., “Can you evaluate the accuracy or distinctiveness of these images on a scale from 0 (*Completely blurry*) to 6 (*Very precise*)?”; Figure 1G). We also asked about the sense of subjective recollection (i.e., feeling of reliving): “When you think about this event do you feel that you are reliving it with all the sensations you had at the time? Are you able to provide many details? And is it so realistic that you feel you are reliving the scene?” We used a film/video metaphor to highlight the nature of recollection: “When you think about this event, imagine that you have rewound the film and are reliving this event as a déjà-vu scene. How do you feel about reliving it with all the sensations you had at the time? Can you rate your feeling of experiencing it on a scale from 0 (*No feeling of reliving*) to 6 (*Very intense feeling*)?” (Figure 1H). Finally, we asked participants about the memory’s personal relevance (e.g., “Was this event important to you? Please indicate your answer on a scale of 0 (*Not at all*) to 6 (*Very important*)”), its frequency of evocation (e.g., “How often do you remember or mention this event on a scale of 0 (*Not at all*) to 6 (*Very often*)”) for past and future events. For future events only, we asked whether they wished them to happen (e.g., “Would you like this event to happen? Please indicate your answer on a scale of 0 (*Not at all*) to 6 (*Very much*)”), and the probability of occurrence (e.g., “Please rate the likelihood of this event happening on a scale of 0 (*Not at all*) to 6 (*Certainly*)”) (Table 2). To ensure that the adolescents made appropriate use of the criteria, we asked them to reformulate the instructions. This procedure was adapted to each participant and repeated until the experimenter was confident that the child understood the judgment criteria.

### Statistical Analyses

Statistical analyses were performed using Statistica Version 10 software (StatSoft, Tulsa, OK, United States). The reported values are means and standard deviations.

Due to the limited number of participants and some non-normally distributed variables (K-S test *p* < 0.05 in one or both groups), we conducted non-parametric analyses (Friedman ANOVAs and Wilcoxon for within comparisons and Mann-Whitney for between comparisons with Z adjusted).

## Results

### General Cognitive Assessment

As expected, Mann–Whitney *U*-test revealed that the ASD group performed more poorly than the TD group on verbal episodic memory (Immediate recall *z* = 2.13; *p* = 0.03, η^2^ = 0.14; Recognition *z* = 2.46; *p* = 0.01, η^2^ = 0.18), and planning (Tower of London, success at first attempt *z* = 2.11; *p* = 0.03, η^2^ = 0.14), but none of the other comparisons including working memory, yielded significant differences (Table 1).

Semantic performance plateaued in both groups (Table 1) confirming the absence of a major deficit in personal semantic knowledge in ASD.

### Personal Event

**FIGURE 2:**
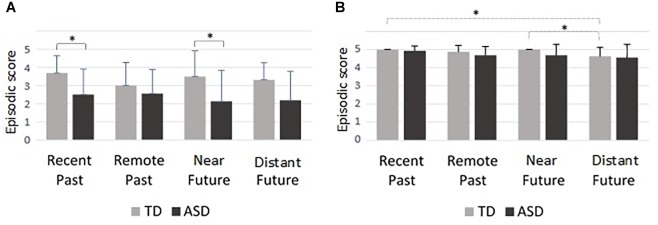
Episodic score: mean performances and standard deviations on **(A)** free recall and **(B)** cued recall for each period according to group. ^∗^*p* < 0.05.

Mann–Whitney *U*-tests on free recall performance revealed significant differences for two periods: recent past (*z* = 2.93, *p* = 0.004, η^2^ = 0.25), near future (*z* = 2.41, *p* = 0.01, η^2^ = 0.18) and a marginally significant effect for the distant future (*z* = 1.95, *p* = 0.056, η^2^ = 0.11). The ASD group produced fewer event memories and projections than the TD group (see Figure 2A).

Mann–Whitney *U*-tests on cued recall performance did not show any differences. However, Friedman ANOVA revealed a significant period effect on performance in the control group (χ^2^ = 13.1, *p* = 0.004, η^2^ = 0.84). The control group reported less details for the distant future period compared to the recent past (*p* = 0.03) and near future periods (*p* = 0.03) (see Figure 2B).

### Emotional Feeling

The analyses of emotion (i.e., valence and arousal) revealed no significant differences between groups (Table 3). However, Friedman ANOVA revealed a significant period effect on arousal in the TD group (χ^2^ = 13.13, *p* = 0.004, η^2^ = 0.84). The arousal associated to memories for the recent past was lower compared to the remote past (*p* = 0.02) and distant future periods (*p* = 0.008). Friedman ANOVA analyses conducted in the ASD group showed a period effect for valence (χ^2^ = 7.72, *p* = 0.05, η^2^ = 0.39). Memories associated with the remote past had a more positive valence than the recent past (*p* = 0.01).

**Table 3:** Mean (SD) emotional feeling and sensory details for each group and each period. Number of details and importance are reported.

			Emotional feeling^#^ (/6)	Color (/6)	Smell (/6)	Sound (/6)	Tactile feeling (/6)	Taste (/6)
	Recent past	ND	4.0 (1.5)	**2.0 (2.0)^∗^**	0.6 (0.7)	1.6 (1.0)	1.1 (0.9)	0.8 (1.3)
		I	4.1 (1.5)	**2.3 (1.8)^∗^**	1.7 (2.2)	3.3 (1.6)	3.0 (2.0)	1.5 (2.2)
ASD	Remote past	ND	4.9 (1.2)	**1.9 (1.6)^∗^**	**0.4 (0.6)^∗^**	1.6 (1.0)	**0.9 (0.7)^∗^**	0.5 (0.6)
		I	4.7 (1.6)	3.7 (1.9)	**1.4 (2.0)^∗^**	**2.8 (1.4)^∗^**	2.7 (2.3)	1.5 (2.1)
	Near future	ND	4.0 (1.7)	2.2 (2.3)	0.4 (0.6)	1.4 (1.3)	1.0 (0.9)	0.5 (0.7)
		I	4.6 (1.3)	2.9 (2.2)	1.3 (1.8)	3.4 (1.9)	3.2 (2.4)	1.6 (2.3)
	Distant future	ND	4.7 (1.2)	2.2 (2.3)	0.9 (1.3)	1.6 (1.0)	1.8 (1.8)	0.4 (0.5)
		I	4.5 (1.3)	3.0 (2.2)	1.7 (2.0)	**4.0 (1.6)^∗^**	3.2 (1.9)	1.8 (2.6)
	Recent past	ND	4.5 (1.4)	3.5 (1.8)	0.6 (0.5)	2.1 (1.4)	1.4 (0.9)	0.4 (0.6)
		I	3.1 (1.3)	3.8 (1.2)	1.6 (1.8)	3.3 (1.1)	3.1 (2.0)	0.9 (1.6)
TD	Remote past	ND	5.0 (0.8)	3.6 (1.6)	1.2 (0.8)	2.5 (1.5)	1.8 (1.2)	0.6 (0.8)
		I	4.5 (1.2)	3.6 (1.1)	2.8 (1.8)	3.8 (1.4)	3.1 (1.7)	1.4 (1.8)
	Near future	ND	4.6 (1.1)	3.3 (2.2)	0.6 (1.0)	2.0 (1.2)	1.8 (1.9)	0.2 (0.4)
		I	4.3 (1.5)	4.0 (1.7)	1.1 (1.7)	3.4 (1.4)	3.0 (2.0)	0.6 (1.5)
	Distant future	ND	4.9 (1.2)	2.6 (1.9)	1.3 (1.3)	2.1 (2.1)	1.6 (1.7)	0.8 (0.9)
		I	4.8 (1.1)	3.7 (1.8)	2.1 (1.7)	2.9 (1.6)	2.3 (2.0)	2.3 (2.2)

*^∗^Significant differences were observed between the ASD and TD groups (in bold), p < 0.05. ^#^Importance refers to arousal for emotions and intensity for sensory details. ASD, participants with autism spectrum disorder; TD, typically developing participants; ND, mean of number of details (SD); I, mean of importance (SD).*

### Sensory Perceptual Details

Analyses on the total number of sensory details showed a significant reduction in the ASD group for the remote past (*z* = 2.74, *p* = 0.006, η^2^ = 0.23). Analyses of each perceptual modality revealed significant differences between the ASD and control group on color for recent past (number *z* = 2.48, *p* = 0.01, η^2^ = 0.19 and intensity *z* = 2.19, *p* = 0.03, η^2^ = 0.15) and for remote past (number *z* = 2.78, *p* = 0.005, η^2^ = 0.24). We also observed differences on smell for remote past period (number *z* = 2.61, *p* = 0.01, η^2^ = 0.19 and intensity *z* = 2.00, *p* = 0.05, η^2^ = 0.12), on sound (intensity for remote past *z* = 2.21, *p* = 0.03, η^2^ = 0.15 and distant future *z* = -2.05, *p* = 0.04, η^2^ = 0.13), and tactile feeling for remote past (number *z* = 2.12, *p* = 0.04, η^2^ = 0.13). Except for sounds for the distant future, the ASD group produced fewer information associated with less intensity than the TD group for all modalities and periods cited above (Table 3).

Friedman ANOVA analyses were conducted within each group on each category of sensory perceptual details. First and concerning the TD group, analyses showed a period effect on both the number and intensity of smell (respectively, χ^2^ = 12.05, *p* = 0.007, η^2^ = 0.75 and χ^2^ = 8.28, *p* = 0.04, η^2^ = 0.44): both scores associated with the near future were reduced compared to the remote past (number *p* = 0.02, intensity *p* = 0.01) and distant future (number *p* = 0.005, intensity *p* = 0.02). Second and concerning the ASD group, analyses showed a period effect on the intensity of colors (χ^2^ = 10.03, *p* = 0.02, η^2^ = 0.58): the intensity of colors associated with the recent past was reduced compared to the remote past (*p* = 0.01). We also observed in this group a period effect on the intensity of sounds (χ^2^ = 10.74, *p* = 0.01, η^2^ = 0.64): sound intensity associated with the remote past was reduced compared to the distant future (*p* = 0.02).

### Recollection and Other Properties

Mann–Whitney comparisons revealed no significant difference for the measures of perspective, personal relevance, wish for it to happen, or probability of occurrence (Table 4). However, the ASD group had lower scores than the TD group on several measures associated to the remote past period: mental imagery (number, *z* = 2.17; *p* = 0.03, η^2^ = 0.14), subjective recollection (*z* = 1.98, *p* = 0.05, η^2^ = 0.12), and frequency of evocation (*z* = 2.3, *p* = 0.02, η^2^ = 0.16). Friedman ANOVA analyses conducted in the TD group showed a period effect on mental imagery (number: χ^2^ = 8.01, *p* = 0.05, η^2^ = 0.39 and accuracy: χ^2^ = 12.24, *p* = 0.007, η^2^ = 0.39). Number of mental imagery associated with recent past was more important than near (*p* = 0.05) and distant (*p* = 0.008) future periods. Accuracy of mental imagery associated with recent past was better than for the remote past (*p* = 0.008) and distant future periods (*p* = 0.008) and accuracy of mental imagery associated with near future was better than distant future period (*p* = 0.05).

**Table 4:** Mean (SD) properties of personal events according to group.

		Mental imagery		Perspective (/3)	Subjective recollection (/6)	Personal relevance (/6)	Frequency of evocation (/6)	Wish for it to happen^#^ (/6)	Probability of occurrence^#^ (/6)
		Number (/6)	Accuracy (/6)						
ASD	Recent past	3.9 (1.7)	4.7 (1.4)	2.4 (0.9)	3.3 (2.0)	3.7 (2.2)	1.9 (1.9)	/	/
	Remote past	**3.1 (1.9)^∗^**	3.9 (1.6)	2.3 (0.9)	**2.6 (1.8)^∗^**	3.8 (1.9)	**2.1 (1.9)^∗^**	/	/
	Near future	3.5 (2.0)	3.5 (2.1)	2.2 (1.0)	3.7 (2.0)	3.5 (2.1)	1.9 (1.9)	3.4 (2.5)	4.8 (1.7)
	Distant future	3.9 (1.8)	4.4 (1.6)	2.1 (1.0)	3.9 (1.7)	3.5 (1.7)	3.2 (2.0)	3.7 (2.3)	4.3 (2.1)
TD	Recent past	4.6 (1.5)	4.9 (1.0)	2.8 (0.6)	4.3 (0.9)	3.8 (1.4)	2.8 (1.6)	/	/
	Remote past	4.5 (1.5)	4.6 (1.0)	2.6 (0.8)	3.7 (1.5)	3.6 (1.5)	3.3 (1.3)	/	/
	Near future	3.9 (1.6)	4.0 (1.5)	2.6 (0.8)	3.4 (1.2)	2.9 (1.5)	2.3 (1.7)	4.3 (1.7)	5.1 (1.3)
	Distant future	3.3 (1.6)	3.8 (1.7)	2.4 (0.9)	3.2 (1.6)	3.3 (1.7)	2.7 (1.7)	4.6 (1.8)	5.5 (0.6)

*^∗^Significant differences were observed between the ASD and TD groups (in bold), p < 0.05. ^#^For future events only. ASD, participants with autism spectrum disorder; TD, typically developing participants.*

## Discussion

The aim of this study was to analyze the properties of past memories and future thinking produced by adolescents with ASD, compared with their TD peers, using a visual cues paradigm. As hypothesized, results revealed difficulty with free recall in the ASD group that contrasted with typical performance on the visually cued task. We found differences between the groups on the total number of sensory details provided only for the remote past period. These differences also appeared when we considered each perceptual modality separately, with the ASD group reporting fewer color, smell, sound, and tactile feeling details and intensity than the TD group. Finally, we did not observe any impairment on the measures of emotion and quality of the experience of recollection, except for number of mental imagery, subjective recollection and frequency of evocation for the remote past.

### Visual Cues in Autobiographical Memory Tasks

Our results showed a significant benefit from visual cues in the production of both past and future episodic autobiographical events. This enhanced performance is in line with the task support hypothesis developed by Bowler et al. (1997), which postulates that performance is better when support is provided at retrieval. Hence, visual cues may be more effective for learning/retrieval, as demonstrated by previous studies that used pictorial prompts for teaching children with ASD (McClannahan and Krantz, 1997; Quill, 1997). AM may be used as a support for social interaction in a social skill program and, for example, ASD participants may use visual cues to share their personal memories.

The impaired performances of participants with ASD on the free recall task were in accordance with their story recall performances (i.e., on the verbal episodic memory test), and mirror previous findings in individuals with ASD (Lind and Bowler, 2010; Brown et al., 2012; Lind et al., 2014a,b). Our data also corroborate the findings of previous studies on future thinking (Terrett et al., 2013; Ciaramelli et al., 2018). In addition, planning difficulties observed in the ASD participants may have contributed to this result. We went beyond them by considering temporal distance and showing impairments of both near that may extend to distant future projections. These impairments may result from difficulty with scene construction, as suggested by Lind et al. (2014b) and, more recently, by Ciaramelli et al. (2018). These authors reported the production of fewer internal details (i.e., episodic), compared with TD controls, but similar numbers of external details (i.e., semantic). Difficulty describing internal states leads to abnormalities in binding experience directly to the self and establishing bonds between the self and others, and consequently, giving coherent meaning to events (Fivush, 2009). Maister and Plaisted-Grant (2011) also suggested that poorer temporal processing abilities in ASD are related to episodic memory impairments. The difficulty accessing episodic AM seemed less pronounced for memories related to the previous and forthcoming summer vacations. Compared with the recent past (restricted to the previous or next day), the more extended vacation period offered a range of possible autobiographical events, facilitating the retrieval of one specific and especially salient moment. Moreover, in contrast to many other studies (Goddard et al., 2014), our task fixed the time period but not the topic, and consequently allowed participants greater flexibility in choosing their personal events, which may have been more closely related to their concerns.

### Sensory Properties

Contrary to our prediction, the episodic memories provided by the participants with ASD contained just as many sensory details as those produced by controls for three periods. These results are in accordance with Crane and Goddard (2008), who did not observe any difference in sensory or emotional information in adults with ASD. This may result, in part, from the use of visual cues for each perceptual modality. However, a lack of details persists for the remote past that may illustrate consolidation difficulties reported by Goddard et al. (2007) and Bon et al. (2012). This reduction is relatively homogeneous and concerned all modalities except taste. Rather surprisingly, however, the recent episodic memories also lacked color details. The adolescents with ASD did mention colors, but fewer than controls. This finding is in accordance with the accounts of some families, who report particular interest in or aversion to some colors and lights in daily life. Some individuals with ASD may have either an obsession with or phobia of colors, as described by Ludlow et al. (2014) in a case study. Hence, they may have an atypical perception of colors that affects the formation/retrieval of memories, even when support is provided. Very few studies have used colored material to study either working memory (see, for example, Vogan et al., 2014) or long-term memory (Massand and Bowler, 2015) in ASD. When Franklin et al. (2008) investigated color memory *per se*, they found impaired performance for colors compared with shapes. Two years later, Franklin et al. (2010) also reported a general reduction in chromatic sensitivity. This atypical sensitivity to color may account for the present results.

### Recollection and Emotional Properties

When our participants with ASD were prompted by visual cues, we did not find any difference in the processing of either the valence or intensity of emotions: they produced memories that were just as positive as those of controls. These results further justify the use of visual cues at retrieval to compensate for the difficulty that individuals with ASD have understanding verbally expressed emotions. Moreover, Maccari et al. (2014) demonstrated that individuals with ASD are able to process positive emotional information embedded in pictures just as well as controls. Our results indicate that this ability can be generalized to familiar autobiographical scenes.

Concerning the other properties, we observed differences between the two groups only for the remote past. The ASD group had reduced mental imagery, subjective recollection and frequency of evocation. Participants with ASD produced memories lacking in details and associated with reduced episodic properties, compared to controls. Once more, this result is in accordance with abnormal forgetting previously reported in ASD. These data replicate those of other experimental studies that used anterograde memory paradigms (Bowler et al., 1997; Souchay et al., 2013; Cooper and Simons, 2018). Our participants’ recollection difficulties may reflect an additional deficit in relational processes, as demonstrated by Bowler et al. (2014) and Gaigg et al. (2015). Individuals with ASD have difficulty binding together the different features that make up an episodic event (Happé and Frith, 2006). Hence, the ASD group may have been successful in recalling some episodic features separately, with the aid of visual cues, but had difficulty binding them together to generate a feeling of reliving. This may be due to weak central coherence, leading to construction, organization, and retrieval difficulties (Happé and Frith, 2006; Bowler et al., 2011), and possibly impacting other abilities such as theory of mind, as suggested recently by Ciaramelli et al. (2018).

Surprisingly, we did not observe the same pattern of performance for projections into the future. Performance was poorer for future versus past periods in the control group for number and accuracy of mental imagery, as previously demonstrated by Abram et al. (2014), thus reducing differences with the ASD group. Hence, the ASD group had an intact feeling of pre-experiencing the future, supporting the notion that the feeling of reliving previous experiences and the pre-experiencing of future events are subtended by partially distinct mechanisms. The feeling of pre-experiencing may have been the product of reasoning based on vividness, the visual perspective adopted during the questionnaire, and personal relevance, as previously demonstrated by D’Argembeau and Van der Linden (2012). All these features were preserved in our participants. The sense of self may be involved to a more limited extent in the ability to elaborate a mental representation associated with future thinking than in the remembering of past autobiographical events.

### Limitations and Perspectives

This work presents certain limits. First, the sample size is relatively small, preventing us from generalizing to the ASD population. In addition, since we had the opportunity to include only boys, inclusion of a group of girls would extend our conclusions to ASD as a whole. Second, our groups do not differ in age but have a wide age range. Given the major influence of age on cognitive development, it would be particularly interesting to investigate the relationship between AM development and other cognitive abilities, such as theory of mind which is impaired in ASD. Third, given the interaction between AM development and social interactions, environment and lifestyle (e.g., family, therapies, activities, etc.), largely neglected in previous studies, it is crucial to consider these factors in future research. Fourth, each personal event was manually transcribed and scored according to a grid coding for five components of episodic memory (i.e., what, where, when, how, who). Scoring was obtained separately by the interviewer and a psychologist until a consensus was reached. In future work, recording verbatim productions would refine the analysis in providing a more detailed investigation of each component. Finally, the interviewer was one of the two coders and was thus not blind to groups. It would be relevant to replicate our results with two coders blind to the diagnoses and verify their inter-rater reliability.

## Conclusion

Our study suggests that AM impairment may result from a combination of a consolidation deficit for the most remote events associated with a binding deficit and demonstrates the relevance of using visual cues to facilitate AM retrieval. These results are in keeping with other studies and may be relevant to other cognitive abilities, as recently suggested by Ciaramelli et al. (2018). This may offer new methodological opportunities for managing ASD. It also shows that some specific properties associated with episodic memories, possibly colors, may be less important than they are to TD people. This raises the issue of the impact of perception on AM, which requires further investigation. In addition, we observed considerable variability, which we could not analyze because of the small size of our sample. Hence, characterizing the different AM profiles should be the next step in studies of cognition in ASD. This could open up new perspectives for cognitive rehabilitation, such as working on AM as the key to social interactions.

## Ethics Statement

Families were given a comprehensive description of the research. The study was approved by the relevant ethics committees, and written consent was obtained from the participants (or their parents, in the case of minors) in line with their guidelines.

## Author Contributions

MA, JLV, FE, and BG-G contributed to the conception and design of the study. MA, JLV, JM, LB, FG, EM, CB, FB-B, and J-MB organized the database. MA, PW, JLV, and BG-G conducted the statistical analysis. MA, PW, and BG-G wrote the first draft of the manuscript. All authors contributed to the manuscript revision, and read and approved the submitted version.

## Conflict of Interest Statement

The authors declare that the research was conducted in the absence of any commercial or financial relationships that could be construed as a potential conflict of interest.

## References

[B1] Abram M. Picard L. Navarro B. Piolino P. (2014). Mechanisms of remembering the past and imagining the future – New data from autobiographical memory tasks in a lifespan approach. *Consc. Cogn.* 29 76–89. 10.1016/j.concog.2014.07.011 25139201

[B2] Albaret J. M. Migliore L. (1999). *Test D’attention Sélective de Stroop*. Paris: ECPA.

[B3] American Psychiatric Association [APA] (2000). *Diagnostic and Statistical Manual of Mental Disorders*, 4th Edn. Washington, DC: American Psychiatric Association.

[B4] Atance C. M. O’Neill D. K. (2005). The emergence of episodic future thinking in humans. *Learn. Motivat.* 36 126–144. 10.1016/j.lmot.2005.02.003

[B5] Bauer P. J. Burch M. M. Scholin S. E. Güler O. E. (2007). Using cue words to investigate the distribution of autobiographical memories in childhood. *Psychol. Sci.* 18 910–916. 10.1111/j.1467-9280.2007.01999.x 17894609

[B6] Bon L. Baleyte J.-M. Piolino P. Desgranges B. Eustache F. Guillery-Girard B. (2012). Growing up with Asperger’s syndrome: developmental trajectory of autobiographical memory. *Front. Psychol.* 3:605. 10.3389/fpsyg.2012.00605 23335906 PMC3542927

[B7] Bowler D. M. Gaigg S. B. Gardiner J. M. (2014). Binding of multiple features in memory by high-functioning adults with autism spectrum disorder. *J. Autism Dev. Dis.* 44 2355–2362. 10.1007/s10803-014-2105-y 24696375

[B8] Bowler D. M. Gaigg S. B. Lind S. E. (2011). “Memory in autism: Binding, self and brain,” in *Researching the Autism Spectrum: Contemporary Perspectives*, eds Roth I. Rezaie P. (Cambridge: Cambridge University Press), 316–346. 10.1017/CBO9780511973918.013

[B9] Bowler D. M. Gardiner J. M. Berthollier N. (2004). Source memory in adolescents and adults with Asperger’s syndrome. *J. Autism .Dev. Dis.* 34 533–542. 10.1007/s10803-004-2548-7 15628607

[B10] Bowler D. M. Matthews N. J. Gardiner J. M. (1997). Asperger’s syndrome and memory: similarity to autism but not amnesia. *Neuropsychologia* 35 65-70. 10.1016/s0028-3932(96)00054-1 8981378

[B11] Brown B. T. Morris G. Nida R. E. Baker-Ward L. (2012). Brief report: making experience personal: internal states language in the memory narratives of children with and without Asperger’s disorder. *J. Autism .Dev. Dis.* 42 441–446. 10.1007/s10803-011-1246-5 21503798

[B12] Bruck M. London K. Landa R. Goodman J. (2007). Autobiographical memory and suggestibility in children with autism spectrum disorder. *Dev. Psychopathol.* 19 73–95. 17241485 10.1017/S0954579407070058

[B13] Ciaramelli E. Spoglianti S. Bertossi E. Generali N. Telarucci F. Tancredi R. (2018). Construction of past and future events in children and adolescents with ASD: role of self-relatedness and relevance to decision-making. *J. Autism. Dev. Dis.* 48 2995–3009. 10.1007/s10803-018-3577-y 29644583

[B14] Cohen M. (2001). *CMS - Echelle Clinique de Mémoire pour Enfant*. Paris: Centre de Psychologie Appliquée.

[B15] Conway M. A. (2005). Memory and the self. *J. Mem. Lang.* 53 594-628.

[B16] Cooper R. A. Simons J. S. (2018). Exploring the neurocognitive basis of episodic recollection in autism. *Psychon. Bull. Rev.* 26 163–181. 10.3758/s13423-018-1504-z 29987766 PMC6424931

[B17] Crane L. Goddard L. (2008). Episodic and semantic autobiographical memory in adults with autism spectrum disorders. *J. Autism. Dev. Dis.* 38 498–506. 10.1007/s10803-007-0420-2 17668308

[B18] Crane L. Goddard L. Pring L. (2009). Specific and general autobiographical knowledge in adults with autism spectrum disorders: the role of personal goals. *Memory* 17 557–576. 10.1080/09658210902960211 19499459

[B19] Crane L. Goddard L. Pring L. (2010). Brief report: self-defining and everyday autobiographical memories in adults with autism spectrum disorders. *J. Autism. Dev. Dis.* 40 383–391. 10.1007/s10803-009-0875-4 19777333

[B20] Crane L. Lind S. E. Bowler D. M. (2013). Remembering the past and imagining the future in autism spectrum disorder. *Memory* 21 157–166. 10.1080/09658211.2012.712976 22901078

[B21] Crane L. Pring L. Jukes K. Goddard L. (2012). Patterns of autobiographical memory in adults with autism spectrum disorder. *J. Autism. Dev. Dis.* 42 2100–2112. 10.1007/s10803-012-1459-2 22322581

[B22] D’Argembeau A. (2015). “Knowledge structures involved in episodic future thinking,” in *Reasoning as memory*, eds Feeney A. Thompson V. A. (New York, NY: Psychology Press), 128–145.

[B23] D’Argembeau A. Van der Linden M. (2012). Predicting the phenomenology of episodic future thoughts. *Consc. Cogn.* 21 1198–1206. 10.1016/j.concog.2012.05.004 22742997

[B24] Fivush R. (2009). Sociocultural perspectives on autobiographical memory. In *The development of memory in infancy and childhood* eds Courage M. L. Cowan N., 2nd Edn. New York, NY: Psychology Press, 283–301.

[B25] Franklin A. Sowden P. Burley R. Notman L. Alder E. (2008). Color perception in children with autism. *J. Autism .Dev. Dis.* 38 1837–1847. 10.1007/s10803-008-0574-6 18449634

[B26] Franklin A. Sowden P. Notman L. Gonzalez-Dixon M. West D. Alexander I. (2010). Reduced chromatic discrimination in children with autism spectrum disorders. *Dev. Sci.* 13 188-200. 10.1111/j.1467-7687.2009.00869.x 20121875

[B27] Gaigg S. B. (2012). The interplay between emotion and cognition in autism spectrum disorder: implications for developmental theory. *Front. Integr. Neurosci.* 6:113. 10.3389/fnint.2012.00113 23316143 PMC3540960

[B28] Gaigg S. B. Bowler D. M. Ecker C. Calvo-Merino B. Murphy D. G. (2015). Episodic recollection difficulties in ASD result from atypical relational encoding: behavioral and neural evidence. *Autism Res.* 8 317–327. 10.1002/aur.144 25630307 PMC4949632

[B29] Goddard L. Dritschel B. Robinson S. Howlin P. (2014). Development of autobiographical memory in children with autism spectrum disorders: deficits, gains, and predictors of performance. *Dev. Psychopathol.* 26 215–228. 10.1017/S0954579413000904 24284059

[B30] Goddard L. Howlin P. Dritschel B. Patel T. (2007). Autobiographical memory and social problem-solving in Asperger syndrome. *J. Autism. Dev. Dis.* 37 291–300. 10.1007/s10803-006-0168-0 16874561

[B31] Goldman S. (2008). Brief report: narratives of personal events in children with autism and developmental language disorders: unshared memories. *J. Autism. Dev. Dis.* 38 1982–1988. 10.1007/s10803-008-0588-0 18512137

[B32] Gottfried J. A. Smith A. P. Rugg M. D. Dolan R. J. (2003). Remembrance of odors past: human olfactory cortex in cross-modal recognition memory. *Neuron* 42 687–695. 10.1016/S0896-6273(04)00270-3 15157428

[B33] Grandin T. (2006). *Thinking in Pictures: And Other Reports from my Life with Autism*. London: Vintage Books.

[B34] Happé F. Frith U. (2006). The weak coherence account: detail-focused cognitive style in autism spectrum disorders. *J. Autism. Dev. Dis.* 36 5–25. 10.1007/s10803-005-0039-0 16450045

[B35] Hassabis D. Maguire E. A. (2007). Deconstructing episodic memory with construction. *Trends Cogn. Sci.* 11 299–306. 10.1016/j.tics.2007.05.001 17548229

[B36] Howe M. L. Courage M. L. (1997). The emergence and early development of autobiographical memory. *Psychol. Rev.* 104 499–523. 10.1037//0033-295x.104.3.499 9243962

[B37] Kleinknecht E. Beike D. R. (2004). How knowing and doing inform an autobiography: relations among preschoolers’ theory of mind, narrative, and event memory skills. *Appl. Cogn. Psychol.* 18 745–764. 10.1002/acp.1030

[B38] Kristen S. Rossmann F. Sodian B. (2014). Theory of own mind and autobiographical memory in adults with ASD. *Res. Autism Spect. Dis.* 8 827–837. 10.1016/j.rasd.2014.03.009

[B39] Lind S. E. Bowler D. M. (2010). Episodic memory and episodic future thinking in adults with autism. *J. Abnor. Psychol.* 119 896–905. 10.1037/a0020631 20853917

[B40] Lind S. E. Bowler D. M. Raber J. (2014a). Spatial navigation, episodic memory, episodic future thinking, and theory of mind in children with autism spectrum disorder: evidence for impairments in mental simulation? *Front. Psychol.* 5:1411. 10.3389/fpsyg.2014.0141 25538661 PMC4256988

[B41] Lind S. E. Williams D. M. Bowler D. M. Peel A. (2014b). Episodic memory and episodic future thinking impairments in high-functioning autism spectrum disorder: an underlying difficulty with scene construction or self-projection? *Neuropsychology* 28 55–67. 10.1037/neu0000005 24015827 PMC3906795

[B42] Lord C. Rutter M. Goode S. Heemsbergen J. Jordan H. Mawhood L. (1989). Autism diagnostic observation schedule: a standardized observation of communicative and social behavior. *J. Autism. Dev. Dis.* 19 185–212. 10.1007/bf02211841 2745388

[B43] Lord C. Rutter M. Le Couteur A. (1994). Autism diagnostic interview-revised: a revised version of a diagnostic interview for caregivers of individuals with possible pervasive developmental disorders. *J. Autism. Dev. Dis.* 24 659–685. 10.1007/bf02172145 7814313

[B44] Losh M. Capps L. (2003). Narrative ability in high-functioning children with autism or Asperger’s syndrome. *J. Autism. Dev. Dis.* 33 239–251. 10.1023/a:1024446215446 12908827

[B45] Ludlow A. K. Heaton P. Hill E. Franklin A. (2014). Color obsessions and phobias in autism spectrum disorders: the case of J.G. *Neurocase* 20 296–306. 10.1080/13554794.2013.770880 23547979

[B46] Lussier F. Guérin F. Dufresne A. (1998). Etude normative développementale des fonctions exécutives: La tour de Londres. *ANAE* 10 42–52.

[B47] Maccari L. Pasini A. Caroli E. Rosa C. Marotta A. Martella D. (2014). Visual search and emotion: how children with autism spectrum disorders scan emotional scenes. *J. Autism. Dev. Dis.* 44 2871–2881. 10.1007/s10803-014-2148-0 24898908

[B48] Maister L. Plaisted-Grant K. C. (2011). Time perception and its relationship to memory in autism spectrum conditions. *Dev. Sci.* 14 1311–1322. 10.1111/j.1467-7687.2011.01077.x 22010891

[B49] Massand E. Bowler D. M. (2015). Atypical neurophysiology underlying episodic and semantic memory in adults with autism spectrum disorder. *J. Autism. Dev. Dis.* 45 298–315. 10.1007/s10803-013-1869-9 23754340

[B50] McCabe A. Hillier A. Shapiro C. (2013). Brief report: structure of personal narratives of adults with autism spectrum disorder. *J. Autism. Dev. Dis.* 43 733–738. 10.1007/s10803-012-1585-x 22767138

[B51] McClannahan L. E. Krantz P. J. (1997). “In search of solutions to prompt dependence: Teaching children with autism to use photographic activity schedules,” in *Environment and Behavior*, eds Baer D. M. Pinkston E. M. (Boulder, CO: Westview), 271–278. 10.4324/9780429039614-29

[B52] McDonnell C. G. Valentino K. Diehl J. J. (2017). A developmental psychopathology perspective on autobiographical memory in autism spectrum disorder. *Dev. Rev.* 44 59–81. 10.1016/j.dr.2017.01.001

[B53] Millward C. Powell S. Messer D. Jordan R. (2000). Recall for self and other in autism: children’s memory for events experienced by themselves and their peers. *J. Autism. Dev. Dis.* 30 15–28. 10.1023/a:1005455926727 10819117

[B54] Mottron L. Burack J. A. Iarocci G. Belleville S. Enns J. T. (2003). Locally oriented perception with intact global processing among adolescents with high-functioning autism: evidence from multiple paradigms. *J. Child Psychol. Psychiatry* 44 904–913. 10.1111/1469-7610.00174 12959498

[B55] Nelson K. (1993). The psychological and social origins of autobiographical memory. *Psychol. Sci.* 4 7–14. 10.1002/wcs.1377 26685796

[B56] Nelson K. Fivush R. (2004). The emergence of autobiographical memory: a social cultural developmental theory. *Psychol. Rev.* 111 486–511. 10.1037/0033-295X.111.2.486 15065919

[B57] Pagulayan K. F. Busch R. M. Medina K. L. Bartok J. A. Krikorian R. (2006). Developmental normative data for the Corsi block-tapping task. *J. Clin. Exp. Neuropsychol.* 28 1043–1052. 10.1080/13803390500350977 16822742

[B58] Perner J. Ruffman T. (1995). Infants’ insight into the mind: how deep? *Science* 308 214–216. 10.1126/science.1111656 15821079

[B59] Picard L. Reffuveille I. Eustache F. Piolino P. (2009). Development of autonoetic autobiographical memory in school-age children: genuine age effect or development of basic cognitive abilities? *Consc. Cogn.* 18 864–876. 10.1016/j.concog.2009.07.008 19733483

[B60] Piolino P. Hisland M. Ruffeveille I. Matuszewski V. Jambaqué I. Eustache F. (2007). Do school-age children remember or know the personal past? *Consc. Cogn.* 16 84–101. 10.1016/j.concog.2005.09.010 16464615

[B61] Quill K. (1997). Instructional considerations for young children with autism: the rationale for visually-cued instruction. *J. Autism. Dev. Dis.* 27 697–714. 9455729 10.1023/a:1025806900162

[B62] Ring M. Gaigg S. B. Altgassen M. Barr P. Bowler D. M. (2018). Allocentric versus egocentric spatial memory in adults with autism spectrum disorder. *J. Autism. Dev. Dis.* 48 2101–2111. 10.1007/s10803-018-3465-5 29380269 PMC5948263

[B63] Schacter D. L. Addis D. R. Hassabis D. Martin V. C. Spreng R. N. Szpunar K. K. (2012). The future of memory: remembering, imagining, and the brain. *Neuron* 76 677–694. 10.1016/j.neuron.2012.11.001 23177955 PMC3815616

[B64] Souchay C. Guillery-Girard B. Pauly-Takacs K. Wojcik D. Z. Eustache F. (2013). Subjective experience of episodic memory and metacognition: a neurodevelopmental approach. *Front. Behav. Neurosci.* 7:212. 10.3389/fnbeh.2013.00212 24399944 PMC3872323

[B65] Stevenson R. A. Siemann J. K. Woynaroski T. G. Schneider B. C. Eberly H. E. Camarata S. M. (2014). Evidence for diminished multisensory integration in autism spectrum disorders. *J. Autism. Dev. Dis.* 44 3161–3167. 10.1007/s10803-014-2179-6 25022248 PMC4224676

[B66] Suddendorf T. Corballis M. C. (1997). Mental time travel and the evolution of the human mind. *Genet. Soc. General Psychol. Monogr.* 123 133–167. 9204544

[B67] Terrett G. Rendell P. G. Raponi-Saunders S. Henry J. D. Bailey P. E. Altgassen M. (2013). Episodic future thinking in children with autism spectrum disorder. *J. Autism. Dev. Dis.* 43 2558–2568. 10.1007/s10803-013-1806-y 23504377

[B68] Tulving E. (1985). How many memory systems are there? *Am. Psychol.* 40 385–398. 10.1037//0003-066x.40.4.385

[B69] Viard A. Chételat G. Lebreton K. Desgranges B. Landeau B. de La Sayette V. (2011). Mental time travel into the past and the future in healthy aged adults: an fMRI study. *Brain Cogn.* 75 1–9. 10.1016/j.bandc.2010.10.009 21093970

[B70] Vogan V. M. Morgan B. R. Lee W. Powell T. L. Smith M. L. Taylor M. J. (2014). The neural correlates of visuo-spatial working memory in children with autism spectrum disorder: effects of cognitive load. *J. Neurodev. Dis.* 6:19. 10.1186/1866-1955-6-19 25057329 PMC4107490

[B71] Wechsler D. (2005). *Echelle d’Intelligence de Wechsler pour Enfants et Adolescents*. Paris: ECPA.

[B72] Welch-Ross M. K. (1997). Mother-child participation in conversation about the past: relationship to the pre-schooler’s theory of mind. *Dev. Psychol.* 33 618–629. 10.1037/0012-1649.33.4.618 9232377

[B73] Wheeler M. A. Stuss D. T. Tulving E. (1997). Toward a theory of episodic memory: the frontal lobes and autonoetic consciousness. *Psychol. Bull.* 121 331–354. 10.1037//0033-2909.121.3.331 9136640

[B74] Williams D. (2010). Theory of own mind in autism: evidence of a specific deficit in self-awareness? *Autism* 14 474–494. 10.1177/1362361310366314 20926458

[B75] Zamoscik V. Mier D. Schmidt S. N. L. Kirsch P. (2016). Early memories of individuals on the autism spectrum assessed using online self-reports. *Front. Psychiatry* 7:79. 10.3389/fpsyt.2016.00079 27199786 PMC4852178

